# Transformations in medical education: A prudential perspective

**DOI:** 10.1002/hcs2.86

**Published:** 2024-03-18

**Authors:** Donald Boudreau, Abraham Fuks

**Affiliations:** ^1^ Faculty of Medicine and Health Sciences Institute of Health Sciences Education McGill University Montreal Québec Canada; ^2^ Department of Medicine Faculty of Medicine and Health Sciences McGill University Montreal Québec Canada

**Abbreviations**
AI: artificial intelligence
CBME: competency based medical education
UGME: undergraduate medical education

## 1 BACKGROUND

A decade ago we asked the question, “Is there something in medicine that is eternal or enduring?” Our aim was to write a manuscript entitled, “That which does not change in medicine.” The publication begins as follows: “The practice of medicine involves continual change, driven by a constant stream of developments in the understanding of biological structure and function relevant to human diseases, and the parallel improvements in pharmacologic and other technological interventions. This change is also driven by evolving social philosophies, ethical trends, and lifestyles.” [1] That preamble reverberates as strongly now as then, perhaps even more so, given the velocity of technological change. When we deliberated on which aspects of medical practice should remain stable, we had few premonitions that implantable chips, robotic surgery, virtual reality, and artificial intelligence (AI) would soon become ubiquitous. The needle has clearly moved, propelled by extraordinary advances in bioengineering, computer and data sciences; major shifts in the governance and organization of clinical practice; and powerful sociocultural trends.

As we explore current transformative developments we are reminded of our earlier conclusions: that certain dimensions of medical practice are, indeed, immutable. Most importantly, the relationship between physician and patient depends on moral obligations, characterized by a compassionate response best described as clinical engagement [2]. The requisite virtues are affective as much as cognitive. The challenge for educators, physicians, and policymakers is to accommodate the benefits of transformational change, both technological and conceptual, whilst remaining true to the fundamental, dyadic clinical relationship. Thus, pedagogic change should welcome innovations but do so with restraint that is, with an attitudinal disposition that is neither cynical nor inhibitory but rather alert and mindful, especially when faced with announcements that a given innovation will solve the problems of an overburdened hospital system. By insisting on a cautious approach we may avoid the pendulum that swings too far, resulting in unintended consequences and costly backtracking to undo the damage of untrammeled enthusiasms.

We consider two illustrative innovations germane to healthcare delivery: one in medical education and one in technology. We try to anticipate and understand impacts and conclude by posing a set of questions that may be useful to those who manage systemic changes.

## 2 A TRANSFORMATION IN MEDICAL EDUCATION

An innovation, unfolding in medical schools world‐wide and often regarded as “transformative,” is competency‐based medical education (CBME). We analyze this trend, relying on a hierarchy of knowledge as a frame of reference.

The historian Jill Lepore, using the metaphor of a filing cabinet with four drawers, proposed a categorization of knowledge [3]. Each drawer contains knowledge of a different kind. The top one is for “mysteries”; the second one is for “facts”; the third is for “numbers”; and the lowest is for “data”. With respect to their epistemologies, she suggests that mysteries are accessible by revelation (thus, discernable by “God”); facts are derived by way of experimental scientific methods or observations (i.e., the product of the natural sciences and the humanities); numbers are entities that can be counted (e.g., measured by statisticians and epidemiologists); and, data are generated by computers (i.e., the product of data science). Each of these categories is potentially valuable to medical practice.

CBME lives, metaphorically speaking, in Lepore's third drawer. Its ontology is reductionist; it views professional development as the accumulation of small quanta of knowledge, accompanied by the accretion of discrete psychomotor skills, which are then presented as stepwise progress in personal abilities. Its epistemology, given the tenacity with which it defines educational outcomes in behaviorally measurable ways, is aligned with psychometrics. The model has been able to mitigate well‐documented deficiencies in the assessments of clinical performance; however, its implementation, theoretical coherence, and axiological foundations have been criticized. Numerous commentators have bemoaned that CBME has monopolized resources, distracted educational leaders, undermined the *in loco parentis* nature of clinical teaching, and, most distressingly, failed to provide a holistic picture of students' evolving professional capabilities [4].

CBME is fraught with controversy. It has become apparent that tensions exist between CBME and the obligation to attend to professional identity formation [5]. A recent commentary attempts to rescue the concept of “competencies” by distinguishing it from “competence” and foregrounding the latter; it argues that competence is multilayered and visualizes the continuum of medical education as existing on three separate levels [6]. The first layer, considered to develop primarily in undergraduate medical education (UGME) and labeled “canonical competence,” represents an amalgam of foundational declarative knowledge with a sprinkling of basic skills. Higher order capabilities, such as creativity, curiosity, adaptability, humility, and leadership, are postponed to later phases of training, in layers encapsulated by the hackneyed phrase, “the art of medicine.” The “art” is supposedly acquired preferentially during residency and in independent practice. This curricular edifice reinforces reductionist aspects of CBME and, in suggesting that it first needs to focus on rote knowledge and skills, undermines arguments for its appropriateness in UGME. There is also a striking disconnect between the wholesale adoption of CBME and the paucity of compelling evidence for its superiority. In Canada, a country that has long promoted CBME, a report of the Royal College of Physicians and Surgeons has confirmed that only a small minority of resident physicians are supportive [7]. Understandably, many jurisdictions have decided to slacken their transition to CBME or to consider adopting a competency‐compatible approach in lieu of a competency‐based model.

The experience with CBME should serve as a note of caution for leaders in the health professions. When faced with changes that are presumed to be salutary, we recommend the following reflections: Might the knowledge represented by this transformation risk overwhelming alternative ways of understanding indispensable to professional or institutional values? Could the change be deforming as much as transforming? To what extent does the adoption of a new concept or innovative tool result in advantages for the adopter (in this illustration, educational managers) and culminate in improved outcomes for the client (in this illustration, clinical teachers and learners)? Perhaps it is time to make haste slowly!

## 3 A TRANSFORMATION IN TECHNOLOGY

The introduction of AI illustrates how new ideas are received, adopted, and translated strategically and pragmatically in medicine. It has been heralded as exciting yet also as disruptive. This technology has unfolded pari passu in educational and clinical domains. Its promises and pitfalls in the former have been summarized [8] and there has been an avalanche of studies, reviews, and commentaries on the scope, risks and benefits of introducing various types of AI to clinically related tasks (e.g., diagnosis, documentation, scheduling, workflow, and simple surgery) and in diverse medical specialities [9]. For purposes of our analysis, we will put aside the specific modes and domains of application and consider the essences of technology, writ large. We turn to Aristotelian philosophical constructs.

The etymology of the word is the Greek *technē* (or *ars*, in Latin)—commonly translated as craft. The ancient Greeks viewed *technē* as the antithesis of *tuchē* (luck). *Tuchē* refers to happenstance that is, events that occur by chance and are not controlled by humans. *Technē* aims to introduce order and predictability. Philosopher Martha Nussbaum notes, “*Technē* …. is a deliberate application of human intelligence to some part of the world, yielding control over *tuchē*; it is concerned with the management of need and prediction and control concerning future contingencies” [10]. Control of contingencies is the cardinal feature. This is highly relevant given that the practice of medicine is awash in contingencies.

Clinical medicine has been defined as: “a science‐using practice that must diagnose and treat illnesses one by one” [11]. That definition helps situate AI in the context of medical practices. AI reinforces the notion that clinical medicine is “science‐using.” It may support physicians' abilities in diagnosing, treating, and prognosticating by reducing the contingencies inherent in these clinical responsibilities. Although AI is founded on databases with an “N” measured in gigabytes, its application in clinical practice must remain resolutely in a mode of “one by one.”

Aristotle distinguished two branches of thought pertinent to human activities in practical domains: fabricating something (e.g., forging a porcelain plate) versus performing something (e.g., navigating a ship). Each activity requires a distinct cognitive disposition. The former requires *technē*—a productive mode with the following characteristics: it identifies features common to a group of cases (i.e., that which is generalizable); it culminates in definable outcomes external to the agent who was involved in the making; it explains phenomena through cause and effect; it is directly teachable; and, it succeeds in creating artifacts through the application of canonical methods. These characteristics of technology, especially the normative aspects, hearken back to the layer of *canonical competence*, as proposed in the retrofit of competence to CBME that we noted earlier.

In contrast to a technological pursuit, performing something corresponds to *phronēsis*. *Phronēsis* has been translated as prudence or practical wisdom. An analogous virtue in Confucianism is *ren* (humanness), moderated by *yi* (justice), which guides a person “to consider contingencies and localized factors” [12]. Practical wisdom allows one to apprehend the singular and its contextual factors (i.e., that which is particular to a case). It is less formulaic than *technē*; requires deliberation about worthwhileness and is thus tied to ethical judgment; is dependent on excellence of character; results in outcomes that are inextricable from the agent; and, is nurtured and transmitted through experiential learning. It has been argued that practices such as medicine and teaching, both focused on an N of 1, are endeavors best understood through the lens of *phronēsis* [13, 14].

It is important to underline that the above concepts have not been defined in crystalline terms and are open to debate. A discussion of philosophical controversies is beyond the remit of this article. The point we wish to make is that medical and pedagogical reasoning involve a choreography between generalization and particularization. These activities must integrate both nomothetic and idiographic approaches, concepts described by the philosopher Wilhelm Windelband [15]. AI, like all technology, cannot escape that dance.

AI will, undoubtedly, help formulate and support the generalizations and precepts routinely used in medicine. Its outputs will improve clinical decision making by rendering diagnostic evaluations, notably those that rely on visual interpretations (e.g., dermatology and radiology), and prognostications increasingly accurate. Time‐honored heuristics that have served physicians well for generations will be fine‐tuned or rendered obsolete by AI. If clinical trials confirm that overall patient outcomes are improved with AI, that is all to the good. However, it is not only the nameless “generic” patient that counts. The singular individual matters …. greatly. Would an AI robot be able to engage with a suffering patient in the manners of a wise clinician? Would a social robot be able to *see* the patient, apprehend their personhood, and accompany them on a healing journey in a comparable three‐dimensional space as would a fellow human being? Randomized controlled studies have demonstrated that large language model‐based systems can generate clinically relevant questions and responses as empathic in tone as those provided by clinicians [16]. Some have interpreted these findings as proof that Chatbot displays “bedside manners” equivalent or superior to those of clinicians [17]. Such conclusions are unwarranted. Interpersonal connections and empathy are complex and multifaceted constructs that cannot be reduced to a question‐and‐answer duet. Considering the nature of practical wisdom can sharpen distinctions between a human and machine clinical encounter.

Two empirical studies on *clinical phronēsis* have revealed some of its characteristics; these include having a capacity for sustaining hope, compassionate rule breaking, creating a bubble of undivided attention, encountering patients in ways that reveal the centrality of their care, and making decisions influenced by embodied perceptions such as gut feelings and intuitions [2, 18]. These, and the development of moral agency, are unlikely to be achievable by robots. AI is destined to become highly desirable for its assistive functions, particularly with respect to generalizations, computations, and pattern recognition. However, in terms of particularization and idiographic approaches within clinical and educational domains, and with respect to moral decision‐making and clinical engagement, we contend that AI will never pass muster. A second reason to make haste … slowly!

## 4 CONCLUSIONS

The “Data, Information, Knowledge, Wisdom” pyramid has been used to represent the intellectual progression from possession of unprocessed data and facts to that of understanding and insight. The origins of this pyramid are uncertain [19]. We find it useful, not least because it positions “wisdom” at the pinnacle. This is, perhaps, more accessible than the “mysteries” visualized by Lepore in the top drawer of her knowledge hierarchy [3]. We propose practical wisdom as an aspirational goal. However, we do not have a magic recipe for its acquisition. Practiced discernment, guided reflection, and longitudinal experiential learning, preferably accompanied by sage mentors, are candidate ingredients.

The following commentary on technology, by Benjamin Chin‐Yee, can help direct necessary reflections. He argues that “… technology does not refer simply to material artifacts but describes a particular way of thinking and interacting with the world; …(it) is not value‐neutral but rather reflects a range of social choices and human values; …(it) does not serve as pure ends to fixed means but instead exists as a continuum of evolving means and ends” [20]. This framing suggests prudence is warranted when one is presented with new technology or new pedagogical models. Critical questions should be posed: (i) Whose interests are served by the innovation, and will anyone be overlooked? (ii) Will any tradition be threatened and if so, is the loss acceptable? (iii) Does its deployment suggest a realignment of core values and beliefs and if so, which are (intentionally or inadvertently) at risk of promotion or demotion? If the guiding principles of clinical medicine find their ethos and telos in the foundational relationship between patient and physician, then we, as healthcare professionals, are obliged to recall that we serve patients and students as the focal point of our duties. We must, therefore, assure that those who have entrusted us with their clinical and pedagogical care will benefit from and not be harmed by our innovations and creativity.

## AUTHOR CONTRIBUTIONS

Both authors participated in the conception and design of this written perspective. Both authors worked on manuscript revisions and contributed to the final version.

## CONFLICT OF INTEREST STATEMENT

The authors declare no conflict of interest.

## ETHICS STATEMENT

Not applicable.

## INFORMED CONSENT

Informed consent is not applicable to this article as no human subjects were involved.

## Data Availability

Data sharing not applicable to this article as no datasets were generated or analyzed in the formulation of this written commentary.
